# Nosocomial Tuberculosis in India

**DOI:** 10.3201/eid1209.051663

**Published:** 2006-09

**Authors:** Madhukar Pai, Shriprakash Kalantri, Ashutosh Nath Aggarwal, Dick Menzies, Henry M. Blumberg

**Affiliations:** * McGill University, Montreal, Quebec, Canada;; † Mahatma Gandhi Institute of Medical Sciences, Sevagram, India;; ‡ Postgraduate Institute of Medical Education and Research, Chandigarh, India;; § McGill University Montreal Chest Institute, Montreal, Quebec, Canada;; ¶ Emory University School of Medicine, Atlanta, Georgia, USA

**Keywords:** tuberculosis, nosocomial transmission, healthcare workers, infection control, hospital acquired infection, occupational exposure, perspective

## Abstract

Most high-income countries implement tuberculosis (TB) infection control programs to reduce the risk for nosocomial transmission. However, such control programs are not routinely implemented in India, the country that accounts for the largest number of TB cases in the world. Despite the high prevalence of TB in India and the expected high probability of nosocomial transmission, little is known about nosocomial and occupational TB there. The few available studies suggest that nosocomial TB may be a problem. We review the available data on this topic, describe factors that may facilitate nosocomial transmission in Indian healthcare settings, and consider the feasibility and applicability of various recommended infection control interventions in these settings. Finally, we outline the critical information needed to effectively address the problem of nosocomial transmission of TB in India.

## Abstract 2

India is well positioned to address the problem of nosocomial tuberculosis transmission.

The risk that *Mycobacterium tuberculosis* can be transmitted from patients with active tuberculosis (TB) to other patients and healthcare workers has been recognized for many years (*1*). The level of risk varies by setting, occupation, patient population, and effectiveness of TB infection control measures (*2**–**5*) but is higher in facilities that manage large numbers of smear-positive TB patients who do not receive rapid diagnosis, isolation, and treatment, particularly in the absence of other infection control measures (*2**–**5*). A hierarchy of control measures, including administrative, engineering, and environmental controls and personal protection measures, has been recommended to reduce nosocomial TB risk (*2**,**3**,**5**,**6*). These recommended measures are implemented by healthcare facilities in high-income countries (*3**,**6*), but given their high cost, few facilities in low-income countries can afford to implement them.

The World Health Organization (WHO) has proposed practical and low-cost interventions to reduce nosocomial transmission in settings where resources are limited (*7*). These recommendations emphasize prompt diagnosis and rapid treatment of TB rather than expensive technologies, such as isolation rooms and respirators. However, despite the widespread implementation of the directly observed therapy, short course (DOTS) strategy, which is internationally recommended, compliance with these simpler guidelines is generally poor in low-income countries (*8*).

In general, the primary focus of national TB programs in high-prevalence, low-income countries is to expand basic DOTS services. Typically, nosocomial transmission is ignored, given countries' limited resources, but several factors illustrate that nosocomial TB must be addressed, even in such areas. First, nosocomial transmission is of concern because it affects not only patients who are exposed but also the healthcare workforce, which could adversely affect healthcare services over time (*7*). Second, transmission of TB can have serious consequences, particularly with multidrug-resistant TB (MDRTB). Several outbreaks in the United States demonstrated the role that hospitals can play as focal points of MDRTB transmission (*9**–**13*), a phenomenon also seen in Europe, South America, South Africa, and Russia (*14**–**16*). These outbreaks can be explosive and associated with high death rates because hospitalized patients are often immunocompromised (*2**,**9*). Therefore, interventions to reduce nosocomial transmission of TB are useful and cost-effective preventive measures to control TB, including MDRTB, particularly in tertiary care settings.

Third, nosocomial TB must be addressed because it can help the healthcare system, particularly the private health sector, improve TB diagnosis and treatment and better align practices with the DOTS strategy. For example, detecting smear-positive TB with microscopy is a key component of the DOTS strategy and an important administrative infection control measure. However, several studies have shown that private practitioners in India tend to underutilize microscopy and rely more on chest radiographs for TB diagnosis (*17**–**19*). Thus, implementation of infection control measures might motivate the private healthcare sector to adopt the DOTS strategy, and implementation of the DOTS strategy may, in turn, enhance infection control.

Fourth, even though low-income countries have fewer resources, ignoring a potential hazard runs contrary to the principles of protecting human health, the cornerstone of health care in any country. Finally, the problem of controlling TB in hospitals is not a problem with TB alone but reflects a problem with infection control in general, which, if improved, could also prevent other infectious diseases (e.g., severe acute respiratory syndrome and avian influenza) that may be nosocomially transmitted. Thus, TB infection control programs can have secondary benefits. Ultimately, preventing outbreaks and protecting patients and staff are in the interests of healthcare facilities. TB infection control is a good starting point for such efforts.

In this article, we focus on India as a case study and review available studies on nosocomial TB, describe factors that facilitate nosocomial transmission, and consider the feasibility of various recommended TB infection control interventions. Finally, we outline critical questions that need to be studied to effectively address nosocomial TB. Although we focus on India, the issues we raise may be applicable to other high-prevalence, resource-limited countries.

## Nosocomial TB in India

India has more TB patients than any other country (*20*) and accounts for one fifth of the world's incident TB cases (*21*); the reported incidence in 2003 was 168 per 100,000 (*20*). Every year, TB develops in nearly 2 million persons in India, and nearly 1 million cases are smear positive; an estimated 40% of the Indian population is latently infected with *M. tuberculosis* (*21*). India's Revised National TB Control Programme (RNTCP) now provides access to DOTS for >85% of the population (*21*). Countrywide coverage is anticipated in 2006 (*22*). This program is the fastest expanding DOTS program in the world and the largest in the world in terms of patients receiving initial treatment (*21*). Outside of the RNTCP, India has a large private health sector that is actively involved in providing TB care (*23**,**24*); almost half of patients with TB in India initially seek care from the private sector (*22*). Thus, because Indian healthcare workers see large numbers of TB patients and because large numbers of TB patients are hospitalized (*25*), the risk for nosocomial exposure is substantial.

Despite the prevalence of TB in India and the expected high probability of nosocomial transmission, little is known about nosocomial TB. In fact, until 2004, no studies on nosocomial TB in India had been published. Table 1 summarizes the results of recent studies on TB among healthcare workers from 3 large tertiary hospitals (*26**–**30*). These studies provide some data on the incidence of active TB (*28**,**29*), prevalence of latent TB infection (*26*), risk factors for active TB (*30*), and annual risk for latent TB infection among healthcare workers (*27*). In addition, another recent study documented person-to-person transmission of TB among hospitalized patients (*31*).

**Table 1:** Recent studies on TB among HCWs in India*

Author, city, year	Setting	Population	Prevalence of latent TB	Incidence of latent TB	Incidence of active TB	Comments
Pai et al., Sevagram, 2005 (*26*)	Rural medical school	726 HCWs, including medical and nursing students (median age 22 y, 62% female) underwent both TST and IGRA	50% positive by TST or IGRA	NA	NA	Prevalence of LTBI was probably underestimated because of nonresponse among senior physicians
Pai et al., Sevagram, 2006 (*27*)	Rural medical school	216 medical and nursing students (median age 21 y) were tested with TST and IGRA; both tests were repeated after 18 mo to document conversions	22% positive by TST, 18% positive by IGRA	5% with TST and IGRA	NA	Annual risk for LTBI was probably underestimated because only students were included in the study
Rao et al., Chandigarh, 2004 (*28*)	Urban tertiary care hospital	701 resident doctors (470 [group 1] were already working at the hospital and 231 [group 2] were newly admitted to the institute); mean age 28 y in group 1 and 26 y in group 2, 81% male	NA	NA	TB developed in 4 of 231 newly admitted residents within 1 y of beginning work, incidence of 17 per 1,000; all except 1 had EPTB	High rate of active TB (mostly EPTB) among HCWs in medical specialties; few cases were bacteriologically confirmed
Gopinath et al., Vellore, 2004 (*29*)	Urban medical school	Retrospective survey to identify HCWs who had TB treatment between 1992 and 2001	NA	NA	125 HCWs underwent TB treatment between 1992 and 2001; 43% of all cases were EPTB, and 5% were MDRTB; incidence of pulmonary TB was 0.35–1.80 per 1,000; incidence of EPTB was 0.34–1.57 per 1,000	EPTB was common; largest number of cases was reported among nurses and nursing students
Mathew et al., Vellore, 2005 (*30*)	Urban medical school	101 HCWs who had had TB disease were compared with 101 randomly selected controls from the same hospital	NA	NA	NA	Body mass index <19 kg/m^2^ and employment in medical wards were independent risk factors for TB disease

*TB, tuberculosis; HCWs, healthcare workers; TST, tuberculin skin test; IGRA, interferon-γ release assay; NA, not available; LTBI, latent TB infection; EPTB, extrapulmonary TB; MDRTB, multidrug-resistant TB.

At a rural medical school hospital in Sevagram, Pai et al. performed the tuberculin skin test (TST) and a whole-blood interferon-γ release assay (IGRA) for 726 healthcare workers (*26*); 50% were positive by either TST or IGRA. Nearly 70% of the participants reported direct contact with sputum smear–positive TB patients. Exposure was particularly high among physicians in training, attending physicians, and nurses. Increasing age and duration of employment were risk factors for latent TB infection. Nurses, nursing students, orderlies, and laboratory staff had higher prevalence of latent infection (*26*). A repeat survey of 216 medical and nursing students in this cohort enabled estimation of the annual risk for latent infection by using TST and IGRA (*27*). When both tests were used, the annual risk for latent TB infection was estimated to be 5% (*27*). The estimated community-based annual risk for infection in India is 1.5% (*32*), so the excess risk of 3.5% may be attributable to nosocomial exposure.

At a tertiary care hospital in Chandigarh, Rao et al. estimated the incidence of active TB among resident physicians (*28*). Among residents already working in the hospital, TB developed in 9 (2%) of 470, for an incidence of 11.2 new cases per 1,000 person-years of exposure. Extrapulmonary disease developed in two thirds of the residents. Overall, this study showed a high rate of TB (predominantly extrapulmonary) among those who worked in medical subspecialties. However, most cases were identified by using clinical criteria, and few were bacteriologically confirmed.

In a retrospective review of healthcare workers who underwent anti-TB treatment in a tertiary care hospital in Vellore, Gopinath et al. identified 125 healthcare workers who had been treated for active TB between 1992 and 2001 (*29*). The annual incidence of pulmonary TB was 0.35–1.80 per 1,000 persons during this period. The annual incidence of extrapulmonary TB was 0.34–1.57 per 1,000. These rates may have been underestimated because only healthcare workers who underwent TB treatment were counted. In this hospital, a case-control study showed that low body mass index and employment in medical wards were risk factors for TB disease among healthcare workers (*30*).

In a molecular epidemiologic study at a TB hospital in Delhi, Bhanu et al. performed DNA fingerprinting on 83 *M. tuberculosis* isolates from patients in 2 adjacent wards (*31*). Of these 83 isolates, 8 strains were grouped into 3 clusters (identical fingerprints) by using IS*6110* restriction fragment length polymorphism and spoligotyping analyses. Within each cluster, epidemiologic data showed overlapping hospitalization periods, which raises the possibility of nosocomial transmission (*31*).

In summary, these studies suggest that nosocomial transmission of TB is a problem in India. The prevalence of latent TB infection and annual risk for TB infection appears to be high even among young healthcare workers. For example, in a hypothetical Indian hospital with 1,000 workers, ≈500 (50%) will likely have latent infection, and ≈25 (5%) of uninfected workers will be newly infected each year. The rate of active disease appears to be exceedingly high in subgroups such as interns, residents, and nurses. The incidences of TB disease and infection are higher than the national averages, which suggests an increased risk for acquiring TB in the hospital setting. For example, the estimated incidence of TB among residents was 10-fold higher than the incidence for the country (*28*).

The predominance of extrapulmonary (mostly pleural) disease among healthcare workers may indicate progression to disease from newly acquired primary infection rather than reactivation of latent TB. Molecular epidemiologic studies suggest that pleural TB is different from other forms of extrapulmonary TB and is associated with the highest fingerprint clustering rate of all forms of TB, which suggests that pleural TB may be an early manifestation of recent infection (*33*). Lastly, although this assumption is based on limited data, nosocomial transmission of TB among hospitalized patients may occur in urban hospitals.

## Factors That May Facilitate Nosocomial Transmission

Several factors may facilitate nosocomial transmission in Indian hospitals, although their relative importance in facilitating transmission is unknown (Table 2). The overwhelming number of TB patients and repeated exposures to smear-positive TB patients are likely to be critical factors. The RNTCP alone starts treatment for >100,000 patients every month (*21*), and thousands more are managed in the private sector (*19**,**22**–**24*). Repeated exposure of trainees is particularly worrisome, given the lack of TB infection control measures at most healthcare facilities. In India, students begin the undergraduate medical program at the age of 17 or 18 years. After an initial classroom-based program in basic sciences, they begin their clinical rotations during years 2 and 3. During this phase of their training, stress is placed on physical examination. Evaluation of the respiratory system, for example, is invariably included in licensure examinations. Because patients with cavitary TB are likely to exhibit signs during a lung exam, TB patients are considered excellent teaching material. Trainees spend considerable time eliciting physical signs in such patients, which results in repeated exposure to patients with infectious TB during trainees' first clinical rotations. This fact may explain the high incidence of infection among them (*27*).

**Table 2:** Factors that may facilitate nosocomial transmission of tuberculosis (TB) in hospitals in India

Area	Factor
Factors that increase risk for nosocomial exposure	Overwhelming numbers of TB patients and repeated exposure to smear-positive TB patients
	Unnecessary or prolonged hospitalization of smear-positive TB patients
	Delays in initiating anti-TB treatment for those with TB
	Poor adherence to treatment, use of suboptimal treatment regimens, and lack of adequate patient support to improve adherence
	Interruptions in supply of TB medications in healthcare facilities
Lack of effective infection-control procedures	Failure to recognize and isolate patients with active pulmonary TB
	Laboratory delays in identification of TB, and poor use of tests such as sputum microscopy to identify infectious TB cases
	Clustering patients with TB with susceptible and vulnerable patients (e.g., HIV-positive patients)
	Lack of HIV testing services and delayed recognition of TB in HIV-infected patients because of atypical presentation and low level of clinical suspicion
	Inadequate respiratory isolation facilities and engineering controls
	Overcrowded hospital wards and outpatient departments
	Poorly ventilated wards and rooms
	Lack of adequate sunlight in hospital wards and departments
	Lack of airborne infection isolation rooms
	Lack of personal protection equipment (e.g., respirators)
	Lack of screening programs to detect and treat TB among healthcare workers
	Lack of commitment on the part of hospitals to invest in infection control programs
	Lack of national guidelines on nosocomial TB tailored to the Indian healthcare environment
Gaps in knowledge and awareness	Lack of awareness about nosocomial TB transmission in healthcare settings in India
	Healthcare workers' belief that nosocomial infection is an occupational hazard that cannot be avoided
	Lack of educational programs on occupational safety and hygiene
	Poor patient education regarding cough etiquette and sputum disposal

Delays in diagnosis and initiation of treatment and failure to separate or isolate patients with smear-positive TB from other patients also contribute to transmission risk. Previous studies in India have shown that diagnostic delays are common, and private practitioners, in particular, tend to underuse sputum microscopy, thereby increasing the probability of missing infectious TB patients (*17**,**19**,**34*). Unnecessary or prolonged hospitalization of TB patients who could have been treated on an ambulatory basis might also contribute to high exposure levels in hospitals. A survey of TB hospitals in India showed that nearly 1 million patients sought treatment in 1999. Approximately 77% of these patients were reported to have undergone sputum examination, and one third of all patients had a diagnosis of TB (*25*). Approximately one third of the hospitals admitted every sputum smear–positive TB patient encountered at their institution.

Several factors might prolong infectiousness of TB patients and thereby facilitate nosocomial transmission. Poor adherence to treatment, lack of continuous drug supply, use of suboptimal treatment regimens, lack of adequate treatment support (e.g., direct observation of therapy [DOT]), and insufficient treatment duration have been reported, particularly in the private sector (*18**,**19**,**24**,**25**,**35**,**36*).

Few hospitals in India have established infection control procedures. Hospitals, especially publicly owned facilities, tend to be crowded, poorly ventilated, and have limited or no facilities for respiratory isolation. Most respiratory care procedures (including sputum collection) are routinely carried out in a general ward setting, rather than in respiratory isolation rooms. Further, few of these hospitals offer routine screening programs to detect and treat TB among healthcare workers.

Previous surveys have identified gaps in knowledge and awareness about TB in healthcare workers in India (*18**,**19**,**24**,**36**,**37*). A survey of 213 nurses in 2 hospitals in Delhi showed that only 67% reported *M. tuberculosis* as the causative organism, and only 22% reported sputum microscopy as the most appropriate way to diagnose TB (*37*). In another survey, only 12% of 204 private practitioners in Delhi reported ordering sputum smears for a patient with suspected TB. For treating TB, 187 physicians used 102 different regimens (*18*). Other surveys have reported similar findings in India (*17**,**19**,**24**,**35**,**36*). Finally, healthcare workers may believe that that they cannot avoid nosocomial infection, which results in resigned acceptance on their part. Since nearly half the Indian population is infected, healthcare workers do not view latent TB infection as a problem. Hence, latent infection is rarely treated, even in high-risk groups such as household contacts and HIV-infected patients (*38**,**39*).

## Implementing TB Infection Control in India

Effective TB infection control in healthcare settings depends on early identification, isolating infected persons, and rapidly and effectively treating persons with TB (*2**,**4**,**5*). In all healthcare settings, a basic TB infection control program should be implemented, as recommended by WHO and other agencies (*2**–**5**,**7*). WHO also recommends developing an infection control plan, educating healthcare workers and patients, improving sputum collection practices, performing triage and evaluation of suspected TB patients in outpatient settings, and reducing exposure in the laboratory (*7*). In the United States, administrative controls (early detection, isolation, and treatment of patients with TB) have been the most effective components of TB infection control programs (*9*).

In India, of all the recommended interventions, implementing administrative controls is likely to be the most feasible and effective strategy. Controls include early detection of patients with infectious TB, isolating or at least segregating those with infectious pulmonary TB from other patients, and rapidly initiating anti-TB treatment, supported by measures to improve adherence (e.g., DOT).

Implementing many of the recommended engineering controls is not feasible in most healthcare facilities because of the high costs of such measures (e.g., negative-pressure isolation rooms). However, separation or segregation of smear-positive TB patients in private or semiprivate rooms or wards with simple mechanical exhaust ventilation (e.g., window fans) could be feasible in some settings, particularly in the private sector and well-funded public hospitals. These measures have been shown to be useful in terminating an outbreak of nosocomial tuberculosis (*9*). This intervention is particularly necessary at centers that manage patients with MDRTB; at such centers, patients with infectious TB must not be admitted to the same wards as patients with HIV infection.

Personal respiratory protection measures (e.g., N95 respirators) are probably not feasible because of the high cost. Respirators may be relatively costly to implement and of limited effectiveness in high-incidence, resource-limited settings (*40*). The use of respirators may have a role in hospitals that manage MDRTB, but more successful and affordable measures include improving natural ventilation through open windows and sunlight. The efficacy of UV germicidal lights is being evaluated in other low-income countries, and results of such studies are needed to determine their value in reducing nosocomial transmission. In developing TB infection control programs, crucial issues are educating healthcare workers about nosocomial TB and measures that can help prevent such transmission, educating patients on cough procedures, and using simple surgical masks on patients with infectious TB (especially if they are not segregated) who are coughing.

Periodic testing of healthcare workers for latent TB and treating those with latent infections who are at high risk for progression to active TB might be feasible in selected settings, particularly among trainees and junior staff (who seem to be disproportionately affected). Screening for latent TB infection with newer, blood-based IGRAs may not be feasible in most settings at this time. Although IGRAs have some advantages over TST, including increased specificity and the ability to discriminate between infection with *M. tuberculosis* and *M. bovis* BCG, they have limited applicability in many resource-limited settings because of the high costs and the need for laboratory infrastructure (*26**,**41*). However, new data suggest that IGRAs hold promise for serial testing of healthcare workers and can overcome some of the limitations of serial tuberculin testing (*27*). A recent study from India showed that in a setting with intensive nosocomial exposure, healthcare workers had strong interferon-γ responses that persistently stayed elevated even after treatment for latent infection (*42*). Persistence of infection or reexposure might account for this phenomenon.

Evaluation of symptomatic healthcare workers for active TB is feasible and should be implemented routinely. In addition to the above measures, hospitals should make every effort to treat TB patients on an ambulatory basis (*25*). If hospitalization is required, every effort should be made to segregate potentially infectious patients from immunocompromised patients, rapidly diagnose and initiate treatment, and discharge patients promptly with DOT on an outpatient basis.

Lastly, efforts should be made to improve the quality of TB care in the private sector through better coordination between the RNTCP and the private sector (*22*). By improving TB diagnosis and treatment practices, smear-positive TB patients are more likely to receive rapid diagnosis and treatment, thereby directly and indirectly reducing the overall transmission in the community and in the nosocomial setting. Such public-private partnerships are currently ongoing in India (*22*), and these programs could address the issue of nosocomial TB.

Who should design and implement TB infection control programs in India? This is a complicated issue because of the variability of healthcare systems in India (e.g., public, private, corporate, nongovernmental, and alternative medical systems). Further, the private sector in India is dominant, diverse, and largely unregulated (*22*). Although a few hospitals have received quality certifications (e.g., ISO 9000), no pressure is on healthcare facilities to get accredited; in fact, India has no national accrediting body. Also, a large proportion of Indians pay for health care with personal funds rather than health insurance.

Given these problems, we cannot envision a simple approach to implementing infection control programs in India. While technical guidance should come from international agencies such as WHO and the International Union Against Tuberculosis and Lung Disease, these guidelines need to be adapted to the Indian context by RNTCP. Ultimately, implementing adequate infection control measures is the responsibility of each healthcare facility. RNTCP may not have the regulatory authority to enforce implementation; however, by partnering with the private sector, RNTCP can improve the quality of case detection and treatment provided in the private sector, which can, by itself, improve infection control.

## Call for Research and Action

Despite India's long and distinguished history of TB research, nosocomial TB has in large part not been addressed by researchers, at least until recently. Although a few studies have been published (*26**–**31*), many more are needed, as summarized in Table 3. A first step is to determine the prevalence of TB among healthcare workers and to evaluate risk factors for nosocomial transmission. In addition, we must assess the availability of resources in India to implement TB infection control measures and to assess what additional resources are needed in areas that have little or no TB infection control programs. India is a vast country with substantial regional variability in resources and expertise. Some healthcare facilities (e.g., private hospitals and medical schools) may have implemented control measures or may have the resources and skills needed to establish effective infection control programs.

**Table 3:** Research needs on nosocomial TB in India*

Area	Specific research questions
Epidemiology and prevalence of disease	What is the prevalence and incidence of latent and active TB among HCWs? Is TB in HCWs more prevalent than in the community?
Molecular epidemiology of transmission of *Mycobacterium tuberculosis* in healthcare settings	What is the likelihood of person-to-person transmission in healthcare settings? How common are nosocomial outbreaks?
Risk factors for exposure to *M. tuberculosis* and risk factors for acquiring LTBI and active disease	What are risk factors for acquiring TB? What are risk factors for patient-to-patient transmission? Why is extrapulmonary disease more common than pulmonary TB among HCWs?
Evaluation of newer diagnostic tools	What is the utility of IGRAs to estimate risk of infection among HCWs? Are IGRAs more accurate, feasible, and cost-effective than TSTs for serial testing of HCWs?
Interventions to reduce nosocomial transmission	What simple, feasible interventions can reduce nosocomial transmission? What is the cost-effectiveness of control programs, and what are long-term benefits to the health system? In HCWs with repeated exposure, what is the long-term efficacy of preventive therapy?
Social, operational, and behavioral issues	What operational and logistic factors increase risk for nosocomial exposure? How common are diagnostic and treatment delays, and how do they affect exposure levels? How does prolonged hospital stay affect risk for nosocomial transmission? How knowledgeable and aware of nosocomial TB are HCWs? What factors affect HCW adherence to interventions that might reduce transmission? How does TB among HCWs affect the healthcare workforce, and how does it affect healthcare delivery? What resources for TB infection control are available in India, and what type of variability exists across healthcare facilities in various states?

*TB, tuberculosis; HCW, healthcare worker; LTBI, latent TB infection; IGRA, interferon-γ release assay; TST, tuberculin skin test.

After assessing the disease prevalence, risk factors, and resources, India must implement effective strategies to reduce nosocomial transmission. To intervene, we will need to know what interventions will and will not work in India. Trials are therefore needed to evaluate relatively simple, feasible interventions and their effectiveness in reducing nosocomial risk. The lessons learned in such trials will be applicable in other resource-limited settings.

In conclusion, healthcare workers are essential in the fight against TB, and their health needs to be protected. India, with its vast human and intellectual capital, nearly countrywide DOTS coverage, and a large, well-funded, successful national TB control program, is well placed to tackle this problem and set an example for other high-prevalence countries.
